# Notes and Miscellany

**Published:** 1911-06

**Authors:** 


### Notes and Miscellany.

---

THIS ISSUE completes the twenty-sixth year of continued service of the TEXAS MEDICAL JOURNAL under one—the original—management.

### A REAL RIP VAN WINKLE

The Strange Case of Dr. Bruno. By F. E. Daniel, M. D. Sold by Von Boeckmann-Jones Company, Austin, Texas. Price, $1.50, or $2.00 with the "Red Back" one year.

Dr. Daniel, the well known editor of the Texas "Red Back" (the *Texas Medical Journal*), has had a quarter of a century of experience as a medical writer, and now, with amazing versatility, he gives us an intensely interesting work of fiction teeming with heart interest. He has blended with the hand of a master, philosophy, religious faith, scientific knowledge, love and romance into an imaginative tale which enthralls the reader from the first page to the close. We were annoyed when compelled to lay it down during our reading, and impatient till we got back to it. The possibility of a synthetic drug inducing prolonged suspended animation is the central theme around which he weaves a weird romance. Well known scientific facts are so deftly threaded among physical impossibilities that the reader must be on his guard lest he dream that astounding discoveries are actually being exemplified and analyzed as demonstrable truths. One feels that the mantle of Jules Verne or Rider Haggard has fallen on worthy shoulders. The doctor who starts to read this book will neglect some of his patients. We know it has thrilled us in a way we have never been thrilled since we first read Ben Hur. The owner will always have a book that he may hand to a friend in assurance that it will be thoroughly enjoyed; but if it is left on the waiting-room table it will surely be stolen.—Dr. A. L. Russell, in *Medical World*, Philadelphia.

Dr. A. L. Lincecum, of El Campo, writes: "Keep the 'Red Back' coming. If it had nothing of interest to me, I would drop it immediately, but I find something of priceless value in each copy."

---

For Sale.—On account of failing health, a $5000 yearly genito-urinary practice in one of Oklahoma's best cities. A snap for right party with little cash or good paper. Write "Doctor," care of Peerless Drug Co., Enid, Okla.

---

Dr. T. B. Selman, Voth, Texas, renews his subscription—three years in advance—and says: "I have been a subscriber ever since I began the practice—twenty-three years ago—and I shall continue to be so long as we are on duty." (More than a quarter of a century.)

---

Dr. Harnam Shah, Punjab, India, writes: "Of all the medical journals I subscribe to, yours is one of the best. I am highly pleased with it. * * * The perusal of Dr. Bruno afforded me great pleasure. It is most entertaining and instructive. I congratulate you upon producing such a book."

---

Wanted.—By a thoroughly capable physician of some years' practice and eighteen months' experience in the United States army hospitals, a salaried position as physician to some mining or smelter corporation in Mexico or New Mexico. Speaks Spanish. Address Dr. C. L., Texas Medical Journal, Austin, Texas.

---

Fellows' Syrupus Hypophosphiticum has been a great favorite with the medical profession for more than a quarter of a century as a tonic, febrifuge and reconstructive. It is one remedy that has held its place and justified the claims made for it. An examination of the formula will indicate its wide range of therapeutic use. Attention is called to the new, unique and very attractive advertisement on third cover page. It is a work of art. Mr. Fellows will be pleased to send literature on request; a postal card will fetch it.

---

Vital Statistics of Texas—(April, incomplete).—The official report of the Registrar shows 4882 births and 2584 deaths; fifty-four sets of twins, one set of triplets. There were thirty-one homicides, one each day and one to spare, and Dallas, the killing center, not heard from. There were fifteen suicides—one every other day and one to spare. Amongst the deaths were one black woman, 115 years old; one white woman, 108 years old; one white woman, 106 years old; one white man, 104 years old; one black woman, 102 years old; one black woman, 101 years old; one white man, 100 years old. In addition to this, there were ten over 90 years old. Tuberculosis (pulmonary) caused 304 deaths, and pneumonia 204. Of the births (4882), 4513 were white; 369 blacks. Of the deaths (2484), 540 were under one year; under five years, 1 to 5—255. Ratio of blacks to whites, not given. Of the births, less than 8 per cent were blacks. Significant!

---

A Good Bismuth Preparation.—After an exhaustive study of the chemical and physical properties of bismuth and its compounds, the chemical experts of Parke, Davis & Co. two or three years ago succeeded in perfecting what many physicians consider the most eligible preparation of the kind—Milk of Bismuth, P.,

D. & Co., a mixture containing the hydrated oxide of bismuth in suspension. The product is stable under all ordinary conditions of temperature and exposure to light and air.

The advantage which Milk of Bismuth, P., D. & Co., possesses over other compounds of the metal is the state of fine subdivision in which the hydrated oxide is presented. This insures its more thorough distribution over the mucous surface of the alimentary canal, upon which it exerts a peculiarly beneficial effect. Its action is not only astringent, but, as some writers have observed, it appears to have a specific effect upon certain lesions, as ulcers, causing them to heal. It is also an antacid and protective, and undoubtedly is mildly antiseptic. Each fluid drachm of Milk of Bismuth, P., D. & Co., represents the bismuth equivalent of five grains of the subnitrate.

---

Sapient President Taft.—An editorial writer in a recent issue of ye Octopus—the *Journal of the A. M. A.*—goes into ecstacies because Mr. Taft said, at a doctors' banquet in Philadelphia recently, that he fully appreciated the value of sanitation and the great economic results obtained by preventing disease. The writer of said article goes into a panegyric, and beslathers Mr. Taft all over with fulsome praise as if he had said something really worth while, but is silent on the fact that William H. is willing for the great public health interest—"the nation's most valuable asset," Roosevelt says—to occupy a pigeonhole (bureau) in the department of the Treasury, or that whose Secretary, in the Cabinet, looks after the health of pigs, for instance. It is as if to say: "Mr. Taft is wise and good. He has confessed that he at last sees the sun, and appreciates its influence on life on this sphere, but says, 'Pshaw! it can't hold a candle to the moon,' meaning that the public health of citizens is of less importance than that of pigs."

If Mr. Taft had the courage of his convictions, as Roosevelt has, he would direct Congress to create a Department of Public Health with a Secretary in the Cabinet, as all enlightened nations have. It is his duty to do it, else he is a hypocrite who defers to the wishes and opinions of the mentally blind, who pretend to see a discrimination against some "school" or pathy, when he knows that every enlightened physician in America favors it, and insists that proper recognition be given the claims of public health as the interest of paramount importance, and so demonstrated in the conquest of disease once so destructive to man.

THE AMARILLO MEETING of the Texas State Medical Association, May 9, 10, 11 (ult.), is said to have been a gratifying success, though not so largely attended as that at Dallas, on account of the remoteness of Amarillo from the center of population and its comparative inaccessibility, etc. I did not attend. The State Association's journal will doubtless give its readers a full account of it, and, as all my Texas readers will get a copy, I leave it to Dr. Taylor.

The new officers for 1911-12 are: President, Dr. D. R. Fly, Amarillo; First Vice-President, Dr. W. H. Freeman, Lockney; Second Vice-President, Dr. A. L. Lincecum, El Campo; Third Vice-President, Dr. J. H. McCracken, Mineral Wells; Fourth Vice-President, Dr. Jno. T. Moore, Houston.

Waco is the place chosen for next convention—May, 1912. The House of Delegates adopted a resolution—by Dr. Holman Taylor—calling on the Legislature to make better laws for the insane and to make provision for them all, and take them out of the jails and poorhouses. This is the outcome of the "Red Back's"

advocacy for many years. The diagnosis of insanity should be made by a commission of medical experts, and not left to a jury of ignorant men. (See our December number.) As to making provision, the insane increase faster than houses can be built or means provided for their care. Enlargements are made, every four years, and the burden of taking care of those already housed —some 5000—amounted to practically a million dollars last year. The thing for the Legislature to do is to pass the bill prepared by the writer of this and introduced into last Legislature to vasectomize all the incurable males discharged or furloughed, thus cutting off the hereditary transmission of the disease, which all superintendents and all properly informed physicians say is the main cause of the never-ending increase. Alcohol caused 46 per cent in twenty-two asylums in sixteen States, as per statistics published in the "Red Back" time and again.

---

TELL YOUR YOUNG DAUGHTERS the meaning of menstruation. In a recent issue of this journal, Dr. Bogart told of a sterile wife who, in ignorance of the meaning of menstruation when a girl, sat down in the snow to stop the hemorrhage. The result Dr. Bogart pictured in forcible language. The following was intended to accompany the recital but came too late. It is too good to be lost. The poor woman, in reply to Dr. B.'s request for permission to use her story, wrote as follows:

"DOCTOR: You will pardon my delay in answering your letter, as I feel that one who could and did so tenderly and so appreciatively seek to lighten my burden deserves better treatment. I have been again in the valley of shadows, and am now so weak that I fear you can not read the letter. Death seemed welcome, at least had little terror, which was selfish of me, for I have a home that is more than home; with love and contentment—material—with flowers, books, pictures, birds—dear birds that come and go and never a cage; a husband who loves me devotedly, and whom I love in full return; friends, the dearest and the most sympathetic, to understand it all, 'she should be happy.' Is the picture complete, with never the patter of little feet, no bumps to kiss away, no little curly heads on pillows, no little stockings to fill in the glad holiday time, no little minds to climb twining through the grand thought of earth?

"Doctor, if the horrors I passed through—the horror that is gripping my heart as I write—will save one else, just one, from the tortures beyond the dreams of Dante, tell it, tell it to the mothers; tell them to tell their daughters, and don't wait; tell it now, now.

"I would not for worlds know that I had caused my bitterest enemy to know a single week of the hell of my girlhood—those years so few, and yet so long, so long, with the trail of sorrow to come, unguessed. I am so weak, and so cold, I must quit writing, but tell the world. It must listen. It must."

---

THE CINCINNATI SANITARIUM— Jno. C. Sheets, President of Directors and Business Manager; Dr. F. W. Langdon, Medical Director. We have received the thirty-seventh annual report of this famous private insane asylum. It makes most interesting and instructive reading. Dr. Langdon will be pleased to send a copy on request. The cases reported were not selected, but taken as they came, and the results of treatment are therefore the more remarkable. Patients on hand, December, 1909, 84—men, 37; women, 47. Received during the year, 235—men, 145; women, 90. Total treated, 319. Discharged, recovered, 70. Improved, 116. Unimproved, 27. Died, 13. Remaining, December 1, 1910, 93. Daily average, 90.14. Monthly rate, 4.07. Recoveries, 29.78.

"THE SANITARIUM IDEA.—The private sanitarium has come to be viewed in modern times as one of the necessary institutions of civilized life.

Insanity is a contingency to be reckoned with—an accident to which we are all more or less liable. It may appear in the course of, or as a sequel to physical illness, such as diseases of the kidneys, heart or blood-vessels, typhoid fever, morbid blood states, accidental injuries, poisoning by various agents, as well as be due to malnutrition, exhaustion, mental overstrain, etc. The patient may suddenly become a source of danger to himself and others.

Such insanity is often temporary, but seclusion and skilled treatment are urgently needed for the time being.

Such patients, in the absence of sanitarium opportunities, must be taken before a court and "charged" with insanity, as if it were an offense. Legal record is made of the case. The sanitarium provides for this class of patients care and treatment without publicity and amid agreeable surroundings."

---

If a urethral discharge persists in spite of active treatment, especially if it be by silver salts, discontinue treatment for a while.—*American Journal of Surgery.*

